# Supplementary material for: An evolutionary functional genomics approach identifies novel candidate regions involved in isoniazid resistance in Mycobacterium tuberculosis
Source: Commun Biol. 2021 Nov 24;4:1322. doi: 10.1038/s42003-021-02846-z (PMC8613195; doi:10.1038/s42003-021-02846-z)
Supplement: Supplementary file 2 — Supplementary Information [file 42003_2021_2846_MOESM2_ESM.pdf]

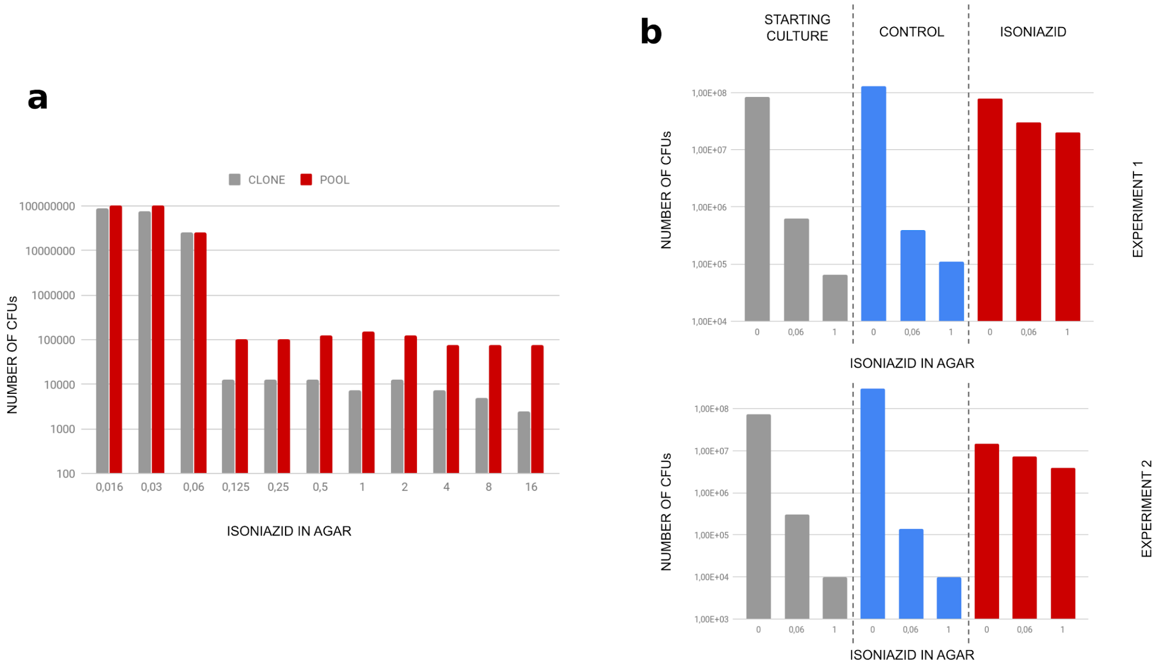

Supplementary Figure 1: a) The pool contains 10 times more isoniazid resistant mutants than its parental clone. **b**) Throughout the experiment, the frequency of isoniazid resistant mutants increased specifically in populations treated with the antibiotic.

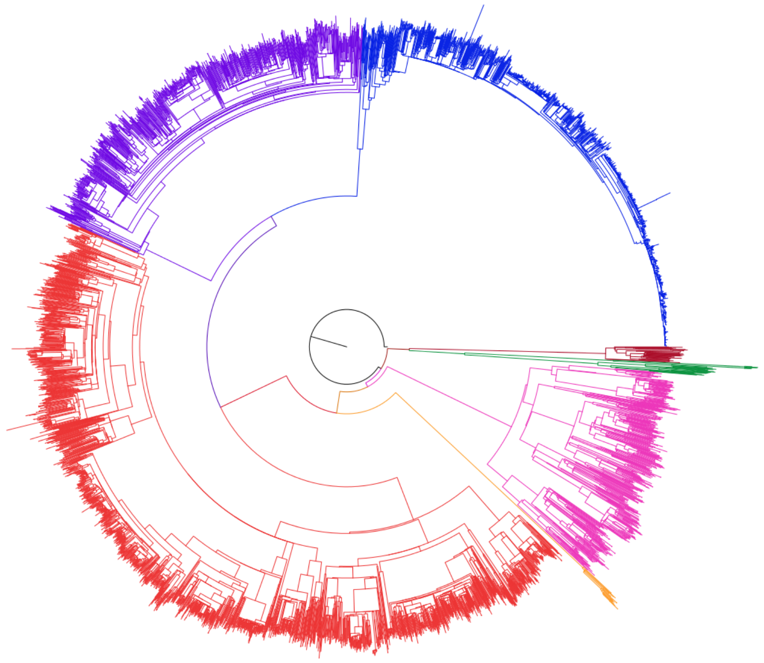

Supplementary Figure 2: Global phylogeny generated with a collated dataset from various sources. Interactive phylogeny can be found at https://itol.embl.de/tree/161111218247308411581608730

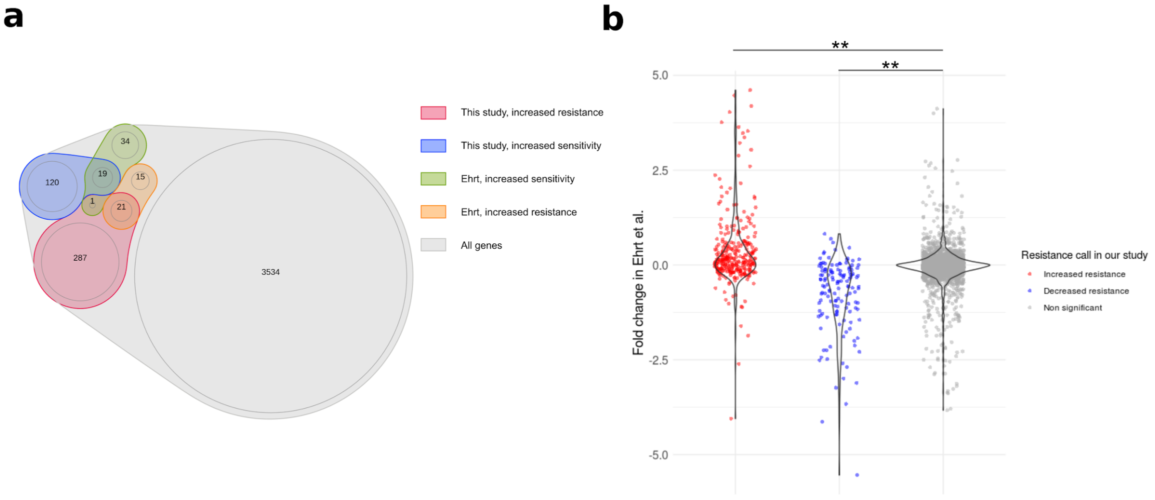

Supplementary Figure 3: a) Coincidence in resistance-altering features detected by functional genomics between our study and that of Ehrt *et al.* **b**) Our resistance-altering features showed similar phenotypes in Xu, W. *et al.* Chemical Genetic Interaction Profiling Reveals Determinants of Intrinsic Antibiotic Resistance in Mycobacterium tuberculosis. *Antimicrob. Agents Chemother.* **61**, (2017).

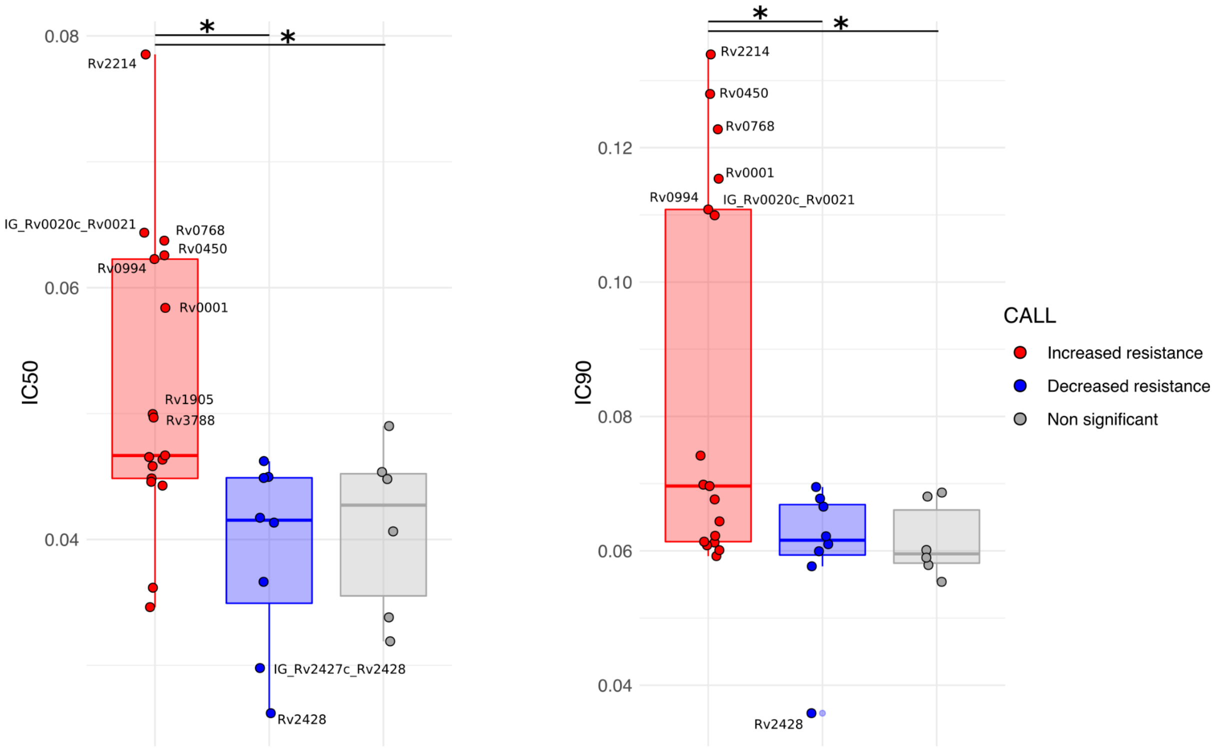

Supplementary Figure 4: Boxplots of IC50 (left) and IC90 (right) values for a collection of candidate gene insertion mutants on a BCG background.

Oligo ID	Sequence	Modification	Purification	Comment
Adap1_Lig	TACCACGACCA	3'AmC7	HPLC	Ligation to gDNA fragments
Adap2_NX_Lig	AGATGTGTATAAGAGACAGTGNNANNANNNTGGTCGTGGTAT	-	HPLC	Ligation to gDNA fragments
Adap2_NX_PCR	GTCTCGTGGGCTCGGAGATGTGTATAAGAGACAG	-	HPLC	Tn-Junction pcr, complementary to Adap2
T7_NX_PCR	TCGTCGGCAGCGTCAGATGTGTATAAGAGACAGCGGGGACTTATCAGCCAACC	-	HPLC	Tn-Junction pcr, complementary to T7 ending sequence

Supplementary Table 1. Oligos used during the experiment for library preparation.

```
begin assumptions;
options deftype=unord;
usertype myCosts (stepmatrix) = 5
0 A T C G
0 0.1 0.1 0.1 0.1
1 0 0.1 0.1 0.1
1 0.1 0 0.1 0.1
1 0.1 0.1 0 0.1
1 0.1 0.1 0.1 0;
typeset * a = myCosts: all;
end;

outgroup MTB_anc;
DerootTrees;
set root=outgroup outroot=monophyl;
RootTrees;
set opt=deltran;
ancstates MTB_anc = 0:all;
assume ancstates=MTB_anc;
log start file=output_paup.txt;
describetrees 1/plot=none chglist=yes;
```

Supplementary Note 1. Assumptions block for the PAUP analysis
