# Supplementary material for: An evolutionary functional genomics approach identifies novel candidate regions involved in isoniazid resistance in Mycobacterium tuberculosis
Source: Commun Biol. 2021 Nov 24;4:1322. doi: 10.1038/s42003-021-02846-z (PMC8613195; doi:10.1038/s42003-021-02846-z)
Supplement: Supplementary file 3 — Description of Additional Supplementary Files [file 42003_2021_2846_MOESM3_ESM.pdf]

Description of Additional Supplementary Files

File name: Supplementary Data 1
Description: Processed TnSeq data and resistance calls for each genomic region.

File name: Supplementary Data 2
Description: Functional and pathway analysis results.

File name: Supplementary Data 3
Description: Accession numbers for the large composite dataset.

File name: Supplementary Data 4
Description: Phylogenetic association data and results.

File name: Supplementary Data 5
Description: Diagnostic resistance mutation list.

File name: Supplementary Data 6
Description: Drug sensitivity of candidate genes insertion mutants.

File name: Supplementary Data 7
Description: Clinical resistance prediction results.

File name: Supplementary Data 8
Description: Accession numbers for the CRyPTIC subset.

File name: Supplementary Data 9
Description: Source data for the main figures.
