# Supplementary material for: An evolutionary functional genomics approach identifies novel candidate regions involved in isoniazid resistance in Mycobacterium tuberculosis
Source: Commun Biol. 2021 Nov 24;4:1322. doi: 10.1038/s42003-021-02846-z (PMC8613195; doi:10.1038/s42003-021-02846-z)
Supplement: Supplementary file 13 — Reporting Summary [file 42003_2021_2846_MOESM13_ESM.pdf]

nature research

Corresponding author(s): Victoria Furió

Last updated by author(s): Oct 20, 2021

Reporting Summary

Nature Research wishes to improve the reproducibility of the work that we publish. This form provides structure for consistency and transparency in reporting. For further information on Nature Research policies, see our Editorial Policies and the Editorial Policy Checklist.

Statistics

For all statistical analyses, confirm that the following items are present in the figure legend, table legend, main text, or Methods section.

n/a	Confirmed	
☐	☒	The exact sample size (*n*) for each experimental group/condition, given as a discrete number and unit of measurement
☐	☒	A statement on whether measurements were taken from distinct samples or whether the same sample was measured repeatedly
☐	☒	The statistical test(s) used AND whether they are one- or two-sided<br>*Only common tests should be described solely by name; describe more complex techniques in the Methods section.*
☒	☐	A description of all covariates tested
☐	☒	A description of any assumptions or corrections, such as tests of normality and adjustment for multiple comparisons
☐	☒	A full description of the statistical parameters including central tendency (e.g. means) or other basic estimates (e.g. regression coefficient) AND variation (e.g. standard deviation) or associated estimates of uncertainty (e.g. confidence intervals)
☐	☒	For null hypothesis testing, the test statistic (e.g. *F*, *t*, *r*) with confidence intervals, effect sizes, degrees of freedom and *P* value noted<br>*Give P values as exact values whenever suitable.*
☒	☐	For Bayesian analysis, information on the choice of priors and Markov chain Monte Carlo settings
☒	☐	For hierarchical and complex designs, identification of the appropriate level for tests and full reporting of outcomes
☒	☐	Estimates of effect sizes (e.g. Cohen's *d*, Pearson's *r*), indicating how they were calculated

Our web collection on statistics for biologists contains articles on many of the points above.

Software and code

Policy information about availability of computer code

Data collection	NextSeq System Suite v2.1.2
Data analysis	We used a custom pipeline available at our GitLab: https://gitlab.com/tbgenomicsunit

For manuscripts utilizing custom algorithms or software that are central to the research but not yet described in published literature, software must be made available to editors and reviewers. We strongly encourage code deposition in a community repository (e.g. GitHub). See the Nature Research guidelines for submitting code & software for further information.

Data

Policy information about availability of data

All manuscripts must include a data availability statement. This statement should provide the following information, where applicable:

- Accession codes, unique identifiers, or web links for publicly available datasets
- A list of figures that have associated raw data
- A description of any restrictions on data availability

TnSeq FASTQ files are available at the European Nucleotide Archive with the project number PRJEB38844. Scripts, datasets and analysis can be found at https://gitlab.com/tbgenomicsunit/ . Strains available under request.

Field-specific reporting

Life sciences study design

All studies must disclose on these points even when the disclosure is negative.

Sample size	Depth of sequencing for the TnSeq experiment was designed so that there would be around 50 reads per insertion to ensure adequate coverage of all insertion sites. Experimental cultures were inoculated with 10 million bacteria, ensuring that on average each insertion mutant would have 100 copies. We allowed bacteria to grow for 13 generations because that is enough to see differences in growth for the different mutants.
Data exclusions	No data excluded
Replication	We compared with similar results from other groups and found good agreement (explained in the paper). We tested 24 insertion mutants for candidate genes for confirmation, comparing them with 6 control mutants
Randomization	The same pool was tested in two independent blocks, in each block we seeded one control and one experimental culture from the same starter culture. Insertion mutants were tested in randomised blocks and with a quality control strain.
Blinding	Blinding was not relevant as all the data collection and analysis is performed by a pipeline

Reporting for specific materials, systems and methods

We require information from authors about some types of materials, experimental systems and methods used in many studies. Here, indicate whether each material, system or method listed is relevant to your study. If you are not sure if a list item applies to your research, read the appropriate section before selecting a response.

Materials & experimental systems

n/a	Involved in the study
☒	☐ Antibodies
☒	☐ Eukaryotic cell lines
☒	☐ Palaeontology and archaeology
☒	☐ Animals and other organisms
☒	☐ Human research participants
☒	☐ Clinical data
☒	☐ Dual use research of concern

Methods

n/a	Involved in the study
☒	☐ ChIP-seq
☒	☐ Flow cytometry
☒	☐ MRI-based neuroimaging
